# Exploring the Relationship between Reactivity and Electronic Structure in Isorhodanine Derivatives Using Computer Simulations

**DOI:** 10.3390/molecules28052360

**Published:** 2023-03-03

**Authors:** Michal Michalski, Slawomir Berski

**Affiliations:** 1 Centre of New Technologies, University of Warsaw, 02-097 Warsaw, Poland; 2 Faculty of Chemistry, University of Wroclaw, 50-383 Wroclaw, Poland

**Keywords:** Diels-Alder, isorhodanine, reactivity, ELF, AIM

## Abstract

The electronic structure and reactivity of 22 isorhodanine (IsRd) derivatives in the Diels–Alder reaction with dimethyl maleate (DMm) were investigated under two different environments (gas phase and continuous solvent ${\mathrm{CH}}_{3}\mathrm{COOH}$), using free Gibbs activation energy, free Gibbs reaction energy, and frontier molecular orbitals to analyze their reactivity. The results revealed both inverse electronic demand (IED) and normal electronic demand (NED) characteristics in the Diels–Alder reaction and also provided insights into the aromaticity of the IsRd ring by employing HOMA values. Additionally, the electronic structure of the IsRd core was analyzed through topological examination of the electron density and electron localization function (ELF). Specifically, the study demonstrated that ELF was able to successfully capture chemical reactivity, highlighting the potential of this method to provide valuable insights into the electronic structure and reactivity of molecules.

## 1. Introduction

The first approach for the hetero-Diels–Alder (hetero-DA) reaction of the thiopyrano[2,3-d]thiazole system was described by I.D. Komaritsa and N.A. Kassab et al., who successfully used 5-arylideneisorhodanines and 5-arylidenethiorhodanines as heterodienes [1,2,3]. Both reagents contain an α, β-unsaturated thiocarbonyl fragment similar to 1-thio-1,3-butadiene, indicating their high reactivity in the [4+2] cycloaddition. The 5-arylideneisorhodanines and 5-arylidenethiorhodanines are prepared via the Knoevenagel reaction of the corresponding 4-thiazolidinethiones [4] and the thionation reaction of 4-thiazolidinones using Lawesson’s reagent or $P_{2}S_{5}$ in anhydrous dioxane [5,6,7]. The green synthesis of 5-arylidene-4-thioxothiazolidines has also been reported by Metwally and performed in PEG-400 under catalyst-free conditions and at room temperature [8]. The dienophile component was initially represented by maleic acid and its derivatives (maleic anhydride, maleinimides), as well as acrylic acid and its derivatives (methyl acrylate, ethyl acrylate, acrylonitrile) [4,6,7]. The list of dienophiles has since been expanded to include cinnamic acids and their amides [9], aroylacrylic and arylidenepyruvic acids [10,11], as well as dimethyl acetylenedicarboxylate [12], propiolic acid, and its ethyl ester [13], acrolein [14], 2-norbornene [15,16], and 5-norbornene-2,3-dicarboxylic acid imides [17]. The use of these new dienophiles has led to the discovery of novel thiopyrano[2,3-d]thiazoles [18].

The Diels–Alder (DA) reaction is classified as a [4+2] cycloaddition due to the interaction of the 4π electron system (the diene structure) with the 2π electron system (the dienophile structure). According to molecular orbital theory, the highest occupied molecular orbital (HOMO) of the diene overlaps with the lowest unoccupied molecular orbital (LUMO) of the dienophile. A key requirement for this reaction is the presence of a strong dienophile with electron-acceptor properties, which decreases the energy difference between the diene’s HOMO and the dienophile’s LUMO. These considerations define the normal electron-demand DA reaction. A conceptually different type of this reaction is the inverse electron-demand DA reaction, which occurs between an electron-rich dienophile and an electron-poor diene. The energy gap between the diene’s LUMO and the dienophile’s HOMO is smaller than between the diene’s HOMO and the dienophile’s LUMO, making the interaction between these two orbitals the most energetically significant stabilizing orbital interaction. According to Conceptual Density Theory, the electronic properties and reactivity of a molecule can be attributed to the energy difference between HOMO and LUMO orbitals, which determine the molecule’s ability to donate or accept electrons. The energy gap between HOMO and LUMO orbitals affects ionization energies and the corresponding changes in charge densities, which are essential in deriving various reactivity parameters, such as electronegativity, hardness, and softness [19,20]. Lately, Domingo and others have applied the ideas on DA and various other reactions, while Shaik has developed a detailed model for organic reactivity that utilizes a valence bond approach and VB diagrams to demonstrate the evolution of different valence bond structures throughout reaction pathways [21,22,23]. In recent years, the Distortion/Interaction Activation Strain model has emerged as a novel approach to understanding reactivity. This model explains the origin of activation barriers in chemical reactions based on the distortion of the reactants and the interaction between the distorted species [24].

The mechanism of DA reaction was investigated using chemical catastrophe theory, which provides a complementary approach to understanding the reaction mechanism based on quantum chemical calculations. By mapping out the reaction pathway and analyzing the electronic and geometric changes that occur during the reaction, catastrophe theory has provided valuable insights into the complex reaction mechanism [25]. Interestingly, to date, no correlation was found between reactivity and the populations of chemical bonds or lone pair electrons calculated through the electron localization function (ELF). Additionally, it should be noted that the reactivity of dienes in DA reaction strongly depends on the electron density on the multiple bonds. The main aim of this work is to present successfully captured chemical reactivity using ELF map in DA reaction, where charge-transfer is the leading term [26]. This demonstrates that ELF method can provide valuable insight into the electronic structure and reactivity of molecules.

The chemical reactivity of the 5-arylidene derivative of 4-sulfanylidene-1,3-thiazolidine-2-one, commonly known as isorhodanine (IsRd), and its isomer, 2-sulfanylidene-1,3-thiazolidine-4-one, commonly named rhodanine, has been previously investigated using quantum chemical methods, in both gas-phase and solvent (acetic acid) models, revealing low energy barrier for IsRd in DA reaction [27]. The current study focuses on analyzing the reactivity between IsRd derivatives with dimethyl maleate (DMm). In this paper, the analysis of geometrical structures and energetic parameters, such as the free Gibbs activation energy and the free Gibbs reaction energy, is followed by a topological analysis of the electron density using AIM analysis proposed by Bader [28] and ELF proposed by Silvi and Savin [29,30]. Finally, the results obtained from these analyses are discussed and summarized.

## 2. Materials and Methods

The geometry optimizations were performed using the density functional theory (DFT) within Gaussian 16 (G16) programme (version C.01) [31] and Minnesota group functional, M06-2X [32], included in G16. The Pople basis set, 6-311+G(d,p) [33,34], with diffuse and polarisation functions were used in these calculations. The 22 chemical groups, i.e., $-{\mathrm{SO}}_{2}{\mathrm{CF}}_{3}$, $-{\mathrm{NO}}_{2}$, $-{\mathrm{SO}}_{3}H$, −CN, $-{\mathrm{CF}}_{3}$, −CHO, −COOH, −NO, −Br, −Cl, −H, −Ph, $-{\mathrm{NHCOCH}}_{3}$, $-{\mathrm{OCH}}_{3}$, −OH, $-N{\left({\mathrm{CH}}_{3}\right)}_{2}$, $-N{\left({\mathrm{CH}}_{2}{\mathrm{CH}}_{3}\right)}_{2}$, $-N{\left(\mathrm{Pr}\right)}_{2}$, $-{\mathrm{NHCH}}_{3}$, $-{\mathrm{NH}}_{2}$, $-S^{-}$, $-O^{-}$ were selected to investigate the influence of functional groups on IsRd reactivity. Optimised structures were characterised by frequency calculation, yielding no imaginary frequencies for reagents and products, and one imaginary frequency for transition states (TSs).

The study considered two distinct environments: the gas phase and acetic acid used as a solvent. To simulate the acetic acid effects, Polarizable Continuum Model (PCM, ε = 6.2528) was used, which was previously validated for organic molecules [35,36,37,38,39,40]. The free Gibbs activation energy ($\Delta G_{a}$) was calculated as a difference between sum of electronic and thermal free energies between TS and optimised structures of the reagents. The free Gibbs reaction energy, $\Delta G_{r}$, has been calculated as difference of sum of electronic and thermal free energies between products and reagents. The energy calculations were performed using gas-phase and PCM models at a temperature of 298.15 K and a pressure of 1 atm.

The electron density, ρ(r), and ELF, η(r), were analyzed to determine chemical bonds using topological analysis. A grid points with a step size of 0.05 bohr was used and calculations were carried out with Multiwfn [41] and TopMod09 [42] packages. The Multiwfn program was also used to calculate the harmonic oscillator model of aromaticity (HOMA) values [43,44] on DFT optimized molecules. Finally, graphical representations of optimized structures and isosurfaces were generated using the Chimera program [45].

## 3. Results and Discussion

**Figure 1:**
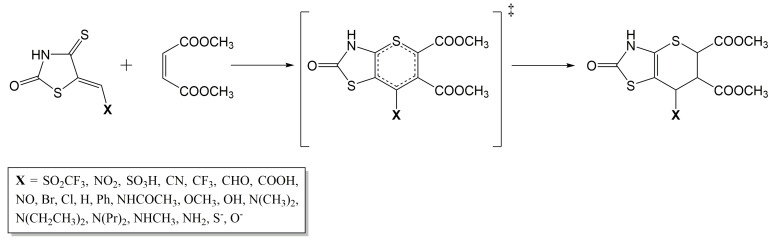
Representation of DA reaction between IsRd derivatives and DMm leading to the formation of the product through a single transition state.

**Figure 2:**
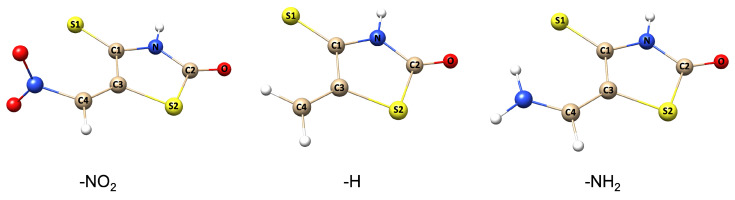
The optimized structures of the $-{\mathrm{NO}}_{2}$, −H, and $-{\mathrm{NH}}_{2}$ IsRd derivatives calculated at the DFT(M06-2X)/6-311+G(d,p) computational level.

The DA reaction is a powerful and well-known method for forming cyclic compounds through the [4+2] cycloaddition of a conjugated diene and a dienophile. The mechanism of DA reaction is characterized by the breaking of three π-bonds and the formation of two single σ-bonds and one π-bond (see Figure 1). The reaction process proceeds through a single transition state, which starts with the formation of the pre-reaction complex. The optimized −H, $-{\mathrm{NO}}_{2}$, and $-{\mathrm{NH}}_{2}$ derivatives of DA reaction are presented in Figure 2. These derivatives, also known as substituents, can have a significant impact on the reactivity and selectivity of DA reaction by modifying the electronic and steric properties of the reacting molecules.

### 3.1. Geometrical Structures and Energetics

The lengths of the C3–C4 and C1–S1 bonds for the optimized structures of the TS are shown in Table 1. The C1–S1 and C3–C4 bond lengths, $r_{\mathrm{opt}}\left(C,S\right)$ and $r_{\mathrm{opt}}\left(C,C\right)$, in the gas-phase study varies between 1.951 ($-O^{-}$) to 2.545 ($-{\mathrm{NO}}_{2}$) Å and 2.205 ($-{\mathrm{NH}}_{2}$) to 2.293 ($-{\mathrm{SO}}_{3}H$) Å, respectively. The values of $r_{\mathrm{opt}}\left(C,S\right)$ are significantly larger than $r_{\mathrm{opt}}\left(C,C\right)$. However, for the $-O^{-}$, $-S^{-}$, $-N{\left({\mathrm{CH}}_{3}\right)}_{2}$, $-N{\left({\mathrm{CH}}_{2}{\mathrm{CH}}_{3}\right)}_{2}$, and $-N{\left(\mathrm{Pr}\right)}_{2}$ calculated $r_{\mathrm{opt}}\left(C,S\right)$ is smaller than $r_{\mathrm{opt}}\left(C,C\right)$. The application of the PCM reveals similarity of $r_{\mathrm{opt}}\left(C,S\right)$ and $r_{\mathrm{opt}}\left(C,C\right)$ values to those obtained in the gas-phase and range 1.974–2.543 Å and 2.181–2.346 Å, respectively. Although obtained C1–S1 and C3–C4 bond lengths differ for $-O^{-}$, $-S^{-}$, $-N{\left({\mathrm{CH}}_{3}\right)}_{2}$, $-N{\left({\mathrm{CH}}_{2}{\mathrm{CH}}_{3}\right)}_{2}$, and $-N{\left(\mathrm{Pr}\right)}_{2}$, the $r_{\mathrm{opt}}\left(C,S\right)$ and $r_{\mathrm{opt}}\left(C,C\right)$ values are in the typical range of carbon–sulphur and carbon–carbon interactions [46] and the selected computational method, DFT(M06-2X)/6–311+G(d,p), can be used to study the reactivity of IsRd derivatives with DMm.

The values of the interaction energy ($E_{\mathrm{int}}$) are presented in Table 2, corrected using the counterpoise correction method [47]. The counterpoise correction method is a computational technique that is used to correct the binding energy of a complex for the basis set superposition error (BSSE), which is an artifact that arises when the monomers of a complex are treated with different basis sets. This method is based on the concept of ghost atoms, which are fictitious atoms that are introduced to cancel out the BSSE by making the monomers of the complex equivalent to the complex. Depending on the derivative, the value of $E_{\mathrm{int}}^{\mathrm{CP}}+\Delta \mathrm{ZPVE}$ ranges between −14.82 ($-{\mathrm{SO}}_{3}H$) and −6.41 (−OH) kcal/mol, indicating that the interaction energy does not show significant deviation across the studied compounds.

The values of the free Gibbs activation energy, $\Delta G_{a}$, and the free Gibbs reaction energy, $\Delta G_{r}$, in the gas phase are presented in Table 1. The value of $\Delta G_{a}$ ranges from 18.47 ($-{\mathrm{SO}}_{2}{\mathrm{CF}}_{3}$) to 28.67 ($-{\mathrm{NH}}_{2}$) kcal/mol, depending on the considered derivative. The value of $\Delta G_{r}$ for meta-directing deactivators (electron withdrawing groups, EWGs) ranges from −22.08 (−CN) to −34.09 ($-{\mathrm{NO}}_{2}$) kcal/mol and is smaller than that obtained for −H, −18.67 kcal/mol. The free Gibbs reaction energy for −Br and −Cl groups is in the range of EWGs. The value of $\Delta G_{r}$ for ortho- and para-directing activators, EDGs, ranges between −25.07 (−Ph) and 23.39 ($-O^{-}$) kcal/mol and is notably higher than for EWGs. In all cases, except for $-O^{-}$ and $-S^{-}$, the product of the reaction is stabilized due to a larger free activation energy on the side of the product, and the probability of the reaction occurring in both directions is small. To conclude this part, the cycloaddition of IsRd with DMm is an exothermic process, and the formation of the product is favored. In the $-O^{-}$ and $-S^{-}$ cases, the cycloaddition of IsRd with DMm is an endothermic process, and the forwards reaction is not favored.

In the presence of the solvent (acetic acid, ${\mathrm{CH}}_{3}\mathrm{COOH}$), the value of $\Delta G_{a}$ ranges between 18.28 ($-{\mathrm{SO}}_{2}{\mathrm{CF}}_{3}$) and 28.79 ($-{\mathrm{NH}}_{2}$) kcal/mol. Interestingly, the analysis performed for the PCM model shows that $\Delta G_{a}$ decreases only for −H, −NO, −CHO, $-{\mathrm{SO}}_{2}{\mathrm{CF}}_{3}$, $-{\mathrm{SO}}_{3}H$, $-O^{-}$, and $-S^{-}$. Thus, the probability of product formation for those derivatives is higher in the solvent than in the gas phase. For the rest of the substituents, the $\Delta G_{a}$ insignificantly increases approximately by 0.53 kcal/mol. The $\Delta G_{r}$ value calculated for the reactions where EWGs have been used ranges between −37.07 ($-{\mathrm{SO}}_{3}H$) and −22.23 (−CN) kcal/mol, and for the reactions with EDGs between −20.50 (−OH) and 16.52 ($-O^{-}$) kcal/mol. Comparison of $\Delta G_{a}$ and $\Delta G_{r}$ in solution and in the gas phase shows that the values were comparable, and acetic acid has a small impact on the energetics of the reaction between IsRd and DMm.

**Figure 3:**
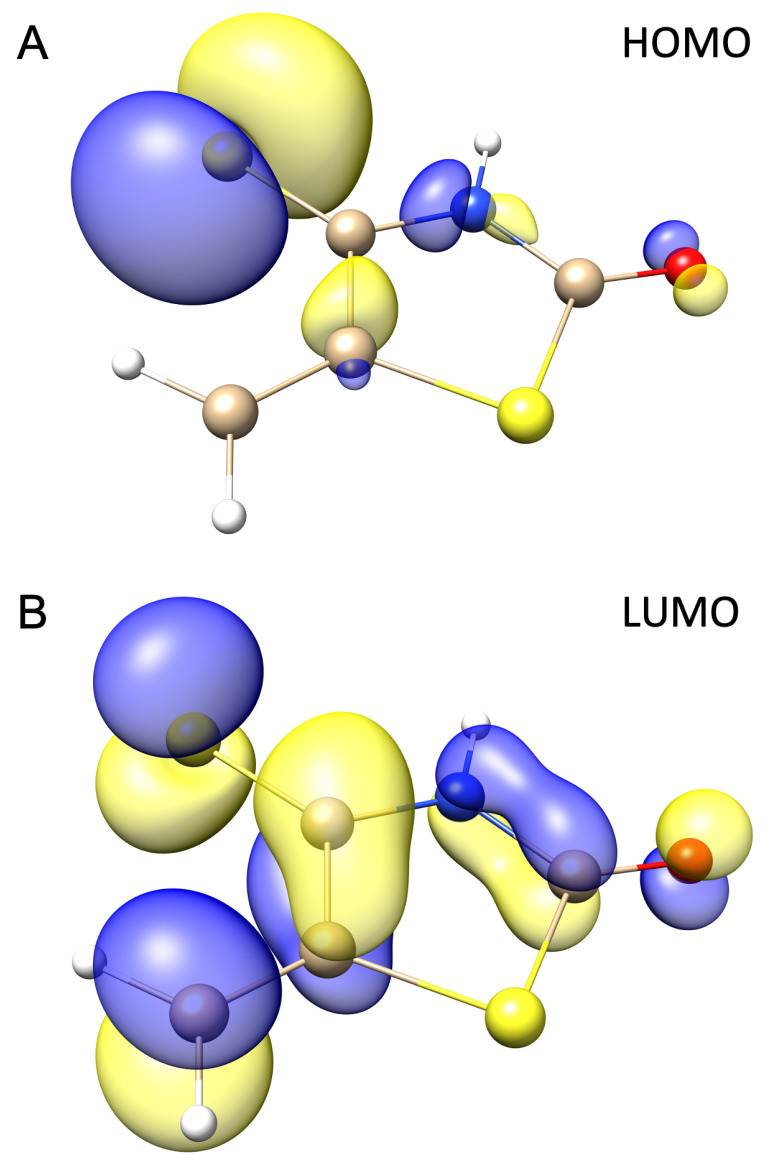
The 3D plot of (**A**) HOMO and (**B**) LUMO for −H IsRd derivative. The isosurfaces is plotted for 0.05 au.

The reactivity and selectivity of a molecule can be well understood by the examination of frontier orbitals [48]. Specifically, HOMO and LUMO can provide insight into the reactivity of IsRd derivatives by determining the energy differences between them for optimized geometries, which is vital in understanding the mechanism of DA reaction. The 3D plots of HOMO and LUMO orbitals for −H are shown in Figure 3, and the energy values of the MOs are compared and stored in Table 2. The energy values of HOMO and LUMO for EWGs range from −8.875 ($-{\mathrm{SO}}_{2}{\mathrm{CF}}_{3}$) to −8.156 ($-{\mathrm{SO}}_{3}H$) eV and from −3.205 ($-{\mathrm{NO}}_{2}$) to −2.322 ($-{\mathrm{SO}}_{3}H$) eV, respectively. Thus, according to the obtained energy values of MOs for −H, one may note a lowering of the total energy of HOMO and LUMO. Different results were obtained for EDGs. The energy values of HOMO and LUMO range between −7.794 ($-{\mathrm{NHCOCH}}_{3}$) and 2.698 ($-O^{-}$) eV and from −2.243 (−Ph) to 3.168 ($-O^{-}$) eV, respectively. Thus, the donating character of those groups results in increasing energy values of frontier MOs.

The character of DA reaction can be understood by looking at the energy differences between HOMO and LUMO orbitals of diene and dienophile. When the energy gap between LUMO of the dienophile and HOMO of the diene is greater than the energy gap between LUMO of the diene and HOMO of the dienophile, it suggests that the reaction has an inverse electronic demand (IED) character. On the other hand, if the energy gap between LUMO of the dienophile and HOMO of the diene is smaller, it suggests that the reaction has a normal electronic demand (NED) character. The energy gaps for EWGs between ${\mathrm{DMm}}_{\mathrm{LUMO}}$-${\mathrm{IsRd}}_{\mathrm{HOMO}}$ are higher than ${\mathrm{IsRd}}_{\mathrm{LUMO}}$-${\mathrm{DMm}}_{\mathrm{HOMO}}$, and suggest the inverse electronic demand (IED) character of DA reaction. Opposite conclusions were obtained for compounds with EDGs due to smaller ${\mathrm{DMm}}_{\mathrm{LUMO}}$-${\mathrm{IsRd}}_{\mathrm{HOMO}}$ to ${\mathrm{IsRd}}_{\mathrm{LUMO}}$-${\mathrm{DMm}}_{\mathrm{HOMO}}$ values. This result indicates the normal electronic demand (NED) character.

### 3.2. The Electronic Properties of Isorhodanine Derivatives

One of the computational methods to investigate the electronic structure of chemical bonds is the topological analysis of electron density [28] and ELF [30], grouped under the umbrella of the quantum chemical topology (QCT) approach [49]. The topological analysis of ELF divides the molecular space into regions that correspond to atomic cores, lone pairs, and chemical bonds, thus having a clear chemical meaning. These spaces are described by basins and critical points and have a one-to-one representation of the expected chemical objects. The critical points are characterized by the number of positive eigenvalues of the Hessian. The local maxima called attractors are critical points where three negative eigenvalues of the Hessian occur. The basins have at least one attractor and may be of two kinds: core basins, C(A), which correspond to the electron density around nuclei; and valence basins, V(A, B, …), which describe valence electrons. The valence basins are characterized by their synaptic order, the number of core basins with which a common boundary is shared. The concept of synapticity has been described by Silvi et al., in reference [50]. Monosynaptic basins are associated with lone pairs, and disynaptic ones with two-center covalent bonds. Polysynaptic basins are characteristic of multicenter bonds.

**Figure 4:**
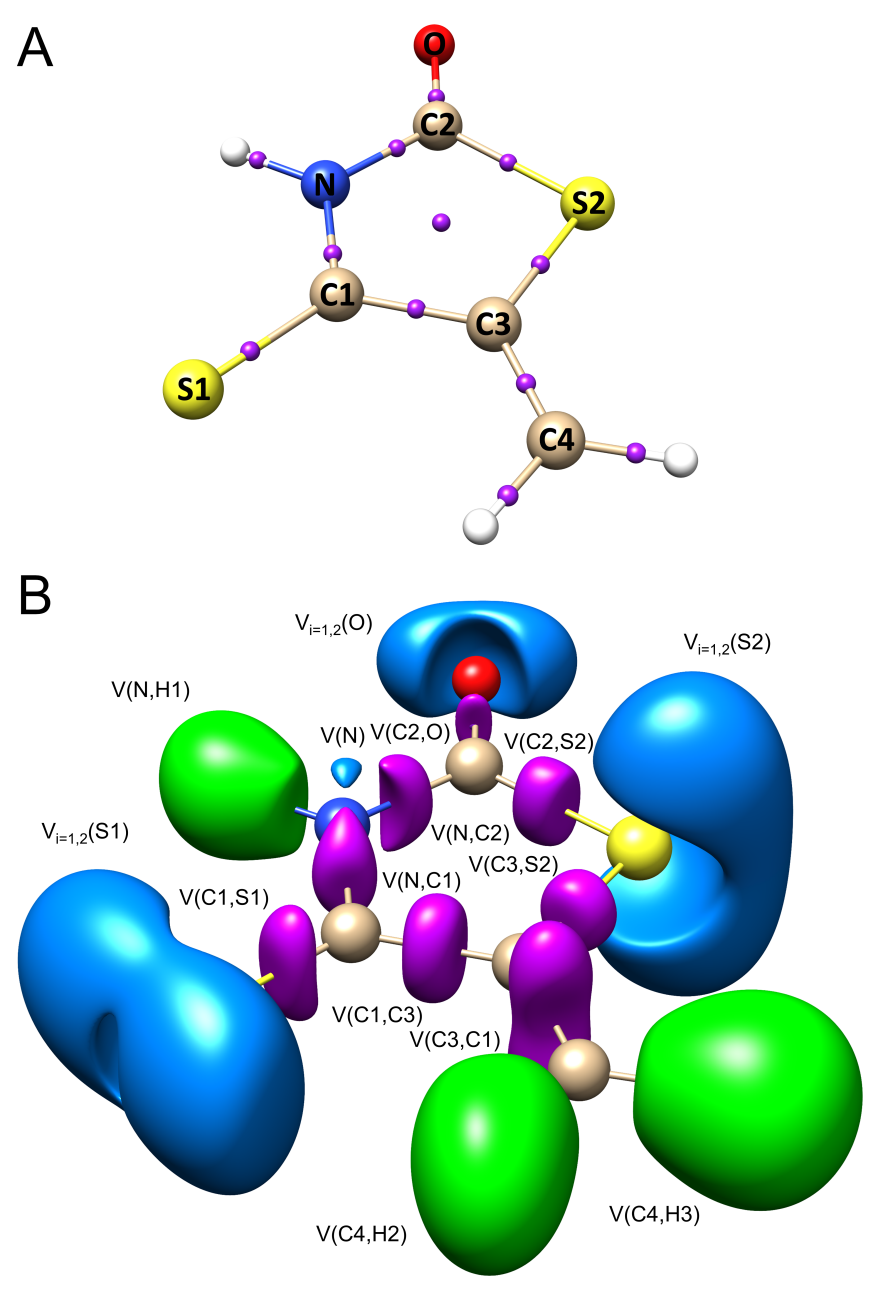
(**A**) The graphical representation of the electron density critical points (purple spheres) for −H IsRd derivative. (**B**) The graphical representation of ELF localization domains (ELF = 0.840) for R = H IsRd derivative. Calculations were carried out at the DFT(M06-2X)/6-311+G(d,p) computational level.

The electronic structure of the heterocyclic ring (see Figure 4A, −H) in IsRd derivatives is investigated from the perspective of topological analysis of η(r) and ρ(r) fields. The values of electron density, $\rho_{\left(3,-1\right)}\left(r\right)$, Laplacian, $\nabla^{2}\rho_{\left(3,-1\right)}\left(r\right)$, delocalization index, DI, and basin populations, $\bar{N}$, are stored in Table 3 and the Supplementary Material (Tables S1–S4). There are eleven critical points of ρ(r) field in the heterocyclic ring: five nuclear critical points (NCPs) (3, −3), five bond critical points (BCPs) (3, −1), and one ring critical points (RCPs) (3, +1). The BCP exhibits a high median value of the electron density and ranges between 0.190 $e/{\mathrm{au}}^{3}$ (C3–S2) and 0.425 $e/{\mathrm{au}}^{3}$ (C2–O). The smallest values were obtained for C2–S2 and C3–S2 which describe the nature of carbon (${\mathrm{sp}}^{2}$) and sulfur (${\mathrm{sp}}^{3}$) interactions in the heterocyclic ring. Interestingly, C1–S1 bond (C: ${\mathrm{sp}}^{2}$, S: ${\mathrm{sp}}^{2}$) has higher $\rho_{\left(3,-1\right)}\left(r\right)$ value than C2–S2 and C3–S2 and equals 0.223 $e/{\mathrm{au}}^{3}$. The median Laplacian of electron density for IsRd derivatives ranges between −1.729 $e/{\mathrm{au}}^{5}$ (N–H1) and 0.086 $e/{\mathrm{au}}^{5}$ (C1–S1). The positive median value of $\nabla^{2}\rho_{\left(3,-1\right)}\left(r\right)$ were only obtained for C1–S1 and C2–O. This indicates that electron density is depleted from the BCP. The Laplacian for other bonds is negative, indicating a concentration of the electron density around BCP may be observed.

The delocalization index is a quantitative measure of the number of electron pairs that are delocalized between two atomic basins. It is often considered to be equivalent to the topological bond order [51]. The median number of electron pairs exchanged between the atomic basin ranges between 0.728 (N–H1) and 1.723 (C1–S1). Thus, for N–H1, C4–H2, N–C2, N–C1, C1–C3, C2–S2, and C3–S2, the DI suggests the presence of a single bond. The highest DI median values were obtained for C1–S1 (DI = 1.723) and C3–C4 (DI = 1.615) bonds. According to the DI interpretation, they may be considered as a double type. The classification of C2–O (DI = 1.340) interaction is ambiguous due to the significantly higher DI than in a single type bonding, 0.728–1.116, but also lower than for a double type, 1.615–1.723.

In the second step, topological analysis has been carried out for ELF. The electronic structure of the heterocyclic ring in all studied derivatives was found to be characterized by five core and five valence attractors. Figure 4B illustrates ELF localization domains for the −H derivative. Calculations performed using DFT(M06-2X)/6-311+G(d,p) shows that the chemical bonds in IsRd are represented by the V(N,H1), V(C4,H2), V(N,C2), V(N,C1), V(C1,C3), V(C1,S1), V(C2,O), V(C2,S2), V(C3,S2), V(C3,C4), and V(C4,R) basins. The R sign reflects the first bonding derivative atom to the C4 carbon atom. All listed ELF basins are disynaptic and represent chemical bonds as a bonding type.

The V(N,H1) and V(C4,H2) attractors describe N–H1 and C4–H2 bonds. The total median population of those basins equal 2.06e and 2.17e, respectively. The calculated $\bar{N}$ values suggest a single character of N–H1 and C4–H2 interactions. The V(N,C2), V(N,C1), V(C1,C3), V(C2,S2), and V(C3,S2) basins describe the chemical bonds in the heterocyclic ring in IsRd. According to the interpretation proposed by Silvi and Savin [30], shared-electron interactions, such as covalent, dative, and metallic bonds occur when there is at least one bond attractor between the core attractors of the atoms involved in the bond. Thus, the localization of those basins proof that the N–C2, N–C1, C1–C3, C2–S2, and C3–S2 interactions are covalent, with shared electron density. The bonding attractors are localized in the regions with high electron localization. The median population equals 2.08e, 2.11e, 2.28e, 1.93e, and 1.77e.

The formal Lewis structure of IsRd presents double character of C1–S1, C2–O, and C3–C4 bonds, which are described by V(C1,S1), V(C2,O), and $V_{i=1,2}\left(C3,C4\right)$ basins. In the EDGs case, such as $-N{\left({\mathrm{CH}}_{3}\right)}_{2}$, $-N{\left({\mathrm{CH}}_{2}{\mathrm{CH}}_{3}\right)}_{2}$, $-N{\left(\mathrm{Pr}\right)}_{2}$, $-{\mathrm{NHCH}}_{3}$, $-{\mathrm{NH}}_{2}$, $-S^{-}$, and $-O^{-}$ the electron density between C3 and C4 atoms is characterized by a single V(C3,C4) basin. The basin population equals 2.55e, 2.46e, and 3.48e, respectively. The topological bond order for C1–S1, C2–O, and C3–C4 equals 1.28, 1.23, and 1.74. For C1–S1 and C2–O bond orders, they are closer to 1, which indicates the single nature of those interactions. The C–O and C–S bonds in $H_{2}C=X$ (X = O, S, Se, Te) have been already studied by Berski et al. [52]. The delocalization of the electron density in $H_{2}C=X$ is dominated by an exchange of electrons between lone pairs of chalcogens for X = (O, S) and delocalization between the lone pairs and core basin for X = (Se, Te). For −H the standard deviations of the $\bar{N}$[V(C1,S1)] and $\bar{N}$[V(C2,O)] are the same and yields 1.16. A high value of the σ confirms the mechanism of fluctuating electron density in the C1–S1 and C2–O bonds. Such results support that the nature of carbon–oxygen and carbon–sulfur interaction should be described by two resonance structures, $C^{+}O^{-}$, $C^{-}O^{+}$ and $C^{+}S^{-}$, $C^{-}S^{+}$, rather than double bonding pairs, C=O and S=O.

**Figure 5:**
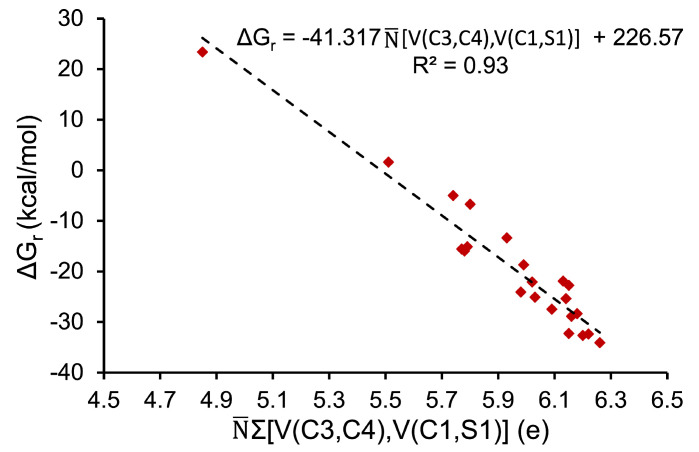
The relationship between the free Gibbs reaction energy, $\Delta G_{r}$ (kcal/mol), and the sum of basin populations, $\bar{N}$∑[V(C3,C4),V(C1,S1)] (e). Linear model is used in the regression analysis.

The hetero-DA reaction is a variant of DA reaction that involves the use of heteroatoms, such as carbonyls and imines, in π-systems. Heteroatoms, defined as atoms other than carbon and hydrogen, play a crucial role in this reaction by introducing new chemical and electronic properties to the reacting molecules. In the hetero-DA reaction, these atoms are utilized to modify the reactivity and selectivity of DA process. In IsRd π-systems are represented by bonding, disynaptic V(C1,S1) and $V_{i=1,2}\left(C3,C4\right)$ basins. The populations of these basins range from 2.26 to 2.68e and 2.59 to 3.63e, respectively, depending on the studied derivative. It is noteworthy that the population of the $V_{i=1,2}\left(C3,\;C4\right)$ basin is affected by the character of nearby chemical substituents, with this effect being more pronounced than for the V(C1,S1) basin. The reactivity of dienes is found to significantly increase when EDG is attached. To investigate this phenomenon, linear regression was utilized to show the relationship between $\Delta G_{r}$ and $\bar{N}$∑[V(C3,C4),V(C1,S1)] using data obtained at the DFT(M06-2X) level. The results of this analysis are presented in Figure 5, which shows that diene reactivity correlates with electron density on both double bonds, with a coefficient of determination, $R^{2}$, equal to 0.93. Unfortunately, a similar correlation based on the free Gibbs activation barriers and $\bar{N}$∑[V(C3,C4),V(C1,S1)] cannot be presented. The highest value of $\bar{N}$∑[V(C3,C4),V(C1,S1)] was observed for $-{\mathrm{NO}}_{2}$ (6.26e: −34.09 kcal/mol) and the lowest for $-O^{-}$ (4.85e: 23.39 kcal/mol). To conclude, topological analysis of ELF reveals a strong impact of electron density on both multiple bonds on diene reactivity in DA reaction.

Finally, the aromaticity of the IsRd derivatives is analyzed using HOMA values [43,44]. This method compares bond lengths between chemical bonds in the studied rings and the idealized bond length in an aromatic ring in benzene. To gain insight into the influence of chemical groups on electron density in the heterocycle ring, we calculated the HOMA index for all studied derivatives at the DFT(M06-2X)/6-311+G(d,p) computational level. All calculations were performed for isolated molecules in the gas phase. The calculated HOMA values are presented in Table 4. The aromaticity of IsRd with −H is intermediate between meta-directing deactivators and ortho- and para-directing activators, and it equals −0.009. For molecules with strong aromatic rings (benzene), the HOMA index should be close to 1.00. Thus, the derivatives with withdrawing groups can be considered slightly antiaromatic, as all HOMA values are clearly smaller than 1.00. When donating groups are attached, HOMA values increase, and the highest values were observed for $-O^{-}$, $-S^{-}$, $-{\mathrm{NH}}_{2}$, $-{\mathrm{NHCH}}_{3}$, $-N{\left(\mathrm{Pr}\right)}_{2}$, $-N{\left({\mathrm{CH}}_{2}{\mathrm{CH}}_{3}\right)}_{2}$, $-N{\left({\mathrm{CH}}_{3}\right)}_{2}$, $-{\mathrm{NHCOCH}}_{3}$. For these substituents, the HOMA index ranges between 0.306 and 0.467, indicating a much higher aromaticity character of the heterocycle ring. Interestingly, −Br and −Cl do not behave as typical withdrawing groups, where the HOMA index is negative, but as donating groups with positive values, 0.049 and 0.066, respectively.

## 4. Conclusions

The reactivity and electronic structure of 22 IsRd derivatives were examined using thermodynamics, frontier molecular orbitals, topological analysis of ELF and electron density, and HOMA. This research enhances our understanding of IsRd reactivity. The main findings can be summarized as follows:

1. The withdrawing and donating substituents produce similar $\Delta G_{a}$ values, and one may assume similar reactivity. However, different conclusions were reached by analyzing $\Delta G_{r}$ values. The free Gibbs reaction energy depends heavily on the chemical group next to the C=C bond. Smaller $\Delta G_{r}$ values were obtained for reactions with EWGs, indicating that the cycloaddition of IsRd with DMm is more favored for derivatives with withdrawing groups. Only for the $-O^{-}$ derivative in the gas phase and PCM model, $\Delta G_{r}$ is positive, making the cycloaddition an endothermic process. Comparison of $\Delta G_{a}$ and $\Delta G_{r}$ in acetic acid and in the gas phase shows that acetic acid has a minimal impact on the energetics of the reaction between IsRd and DMm.
2. The analysis of frontier DFT orbitals performed on molecules with EWGs shows a decrease in the electronic energy of HOMO and LUMO orbitals, indicating that a NED character of DA reaction is expected. For EDGs, the energy of frontier MOs increases, resulting in an IED type of cycloaddition reaction.
3. Topological analysis of ρ(r) shows positive values of the Laplacian for BCPs only for the C1–S1 and C2–O bonds, indicating a depletion of electron density around BCP. The Laplacian values for other bonds are negative, indicating a concentration of electron density around BCP. Thus, C1–S1 and C2–O bonds exhibit a different nature than observed for other covalent bonds where both atoms share electron density. The DI values suggest a single bond type for N–H1, C4–H2, N–C2, N–C1, C1–C3, C2–S2, C3–S2 and a double type for C1–S1 and C3–C4 bonds. The bonding type for C2–O cannot be interpreted by means of DI.
4. Topological analysis of ELF partly confirms the results based on electron density. The topological bond orders for C1–S1 and C2–O are 1.28 and 1.23, respectively, thus one may consider these interactions to be of a single nature. Analysis of σ suggests that the $C^{+}O^{-}$, $C^{-}O^{+}$ and $C^{+}S^{-}$, $C^{-}S^{+}$ representations for the carbon–oxygen and carbon–sulphur interactions, instead of the classical C=O and S=O formulas, provide a better description of the bond nature.
5. The most intriguing discovery was obtained by analyzing the relationship between $\Delta G_{r}$ and the basin populations for the C=S and C=C bonds in the IsRd molecule. The analysis of ELF was performed on isolated molecules. According to the proposed DA reaction mechanism, the electron density from these bonds is distributed to the regions of the two new C–S and C–C bonds that are formed between the IsRd and DMm molecules. The regression analysis applied to this relationship shows that the values of the sum of the populations for the V(C1,S1) and $V_{i=1,\;2}\left(C3,\;C4\right)$ basins, $\bar{N}$∑[V(C3, C4), V(C1, S1)], correlates with the value of the free Gibbs reaction energy. Large negative values of $\Delta G_{r}$ correspond to large values of the sum of basin populations. The favorability of the studied DA reaction, which is associated with a rearrangement of chemical bonds and electron density, is high when the C=S and C=C bonds are better saturated with electron density.
6. The HOMA values indicate low aromaticity of the IsRd ring.

## Figures and Tables

**Table 1:** The values of the selected bond lengths, $r_{\mathrm{opt}}$ (Å), between the interacting atoms in IsRd⋯DMm TS derivatives, the free Gibbs activation energy, $\Delta G_{a}$ (kcal/mol), and the free Gibbs reaction energy, $\Delta G_{r}$ (kcal/mol), calculated for DA reaction with DMm.

R	Gas Phase				Solvent (Acetic Acid)			
	$r_{\mathrm{opt}}$(C,C)	$r_{\mathrm{opt}}$(C,S)	$\Delta G_{a}$	$\Delta G_{r}$	$r_{\mathrm{opt}}$(C,C)	$r_{\mathrm{opt}}$(C,S)	$\Delta G_{a}$	$\Delta G_{r}$
$-{\mathrm{SO}}_{2}{\mathrm{CF}}_{3}$	2.284	2.527	18.47	−32.66	2.291	2.520	18.28	−34.02
$-{\mathrm{NO}}_{2}$	2.259	2.545	19.17	−34.09	2.264	2.543	19.73	−34.24
$-{\mathrm{SO}}_{3}H$	2.293	2.530	22.72	−32.37	2.296	2.528	18.99	−37.07
−CN	2.258	2.437	22.34	−22.08	2.263	2.442	22.76	−22.23
$-{\mathrm{CF}}_{3}$	2.279	2.503	20.97	−32.25	2.287	2.503	21.60	−32.63
−CHO	2.210	2.506	19.47	−24.07	2.218	2.499	19.17	−25.49
−COOH	2.236	2.526	19.52	−27.48	2.243	2.525	19.71	−27.97
−NO	2.232	2.463	21.05	−25.35	2.245	2.458	19.62	−27.16
−Br	2.255	2.499	23.04	−28.85	2.263	2.497	23.36	−29.19
−Cl	2.244	2.494	22.84	−28.34	2.252	2.492	23.05	−28.71
−H	2.216	2.458	24.78	−18.67	2.213	2.473	20.48	−23.14
−Ph	2.283	2.451	20.27	−25.07	2.292	2.448	20.49	−25.49
$-{\mathrm{NHCOCH}}_{3}$	2.220	2.377	26.49	−13.35	2.241	2.364	26.94	−14.12
$-{\mathrm{OCH}}_{3}$	2.245	2.416	22.40	−21.89	2.257	2.396	23.28	−20.00
−OH	2.239	2.421	22.49	−22.75	2.246	2.401	23.44	−20.50
$-N{\left({\mathrm{CH}}_{3}\right)}_{2}$	2.271	2.254	20.52	−15.09	2.334	2.175	21.42	−11.35
$-N{\left({\mathrm{CH}}_{2}{\mathrm{CH}}_{3}\right)}_{2}$	2.284	2.247	19.76	−15.94	2.346	2.176	20.86	−12.82
$-N{\left(\mathrm{Pr}\right)}_{2}$	2.279	2.248	19.88	−15.57	2.341	2.179	20.59	−12.65
$-{\mathrm{NHCH}}_{3}$	2.208	2.314	27.95	−4.99	2.250	2.252	28.26	−3.90
$-{\mathrm{NH}}_{2}$	2.205	2.338	28.67	−6.72	2.236	2.281	28.79	−6.02
$-S^{-}$	2.293	2.132	26.91	1.61	2.326	2.192	26.50	−6.71
$-O^{-}$	2.064	1.951	28.37	23.39	2.181	1.974	27.13	16.52

**Table 2:** The values of the interaction energy, $E_{\mathrm{int}}$ (kcal/mol), corrected by the difference of the zero-point vibrational energies, ΔZPVE (kcal/mol), and the basis set superposition error, BSSE (kcal/mol), for the pre-reaction complexes formed by the IsRd derivatives with DMm, the HOMO and LUMO energy orbitals (eV) for IsRd derivatives, and the difference of the energies (eV) of the LUMO-HOMO and HOMO-LUMO orbitals.

R	$E_{\mathrm{int}}$	BSSE	ΔZPVE	$E_{\mathrm{int}}^{\mathrm{CP}}+\Delta \mathrm{ZPVE}$	HOMO	LUMO	${\mathrm{DMm}}_{\mathrm{LUMO}}$-${\mathrm{IsRd}}_{\mathrm{HOMO}}$	${\mathrm{IsRd}}_{\mathrm{LUMO}}$-${\mathrm{DMm}}_{\mathrm{HOMO}}$
$-{\mathrm{SO}}_{2}{\mathrm{CF}}_{3}$	−12.36	3.18	0.76	−8.42	−8.875	−3.081	8.026	6.433
$-{\mathrm{NO}}_{2}$	−11.44	2.65	1.00	−7.79	−8.792	−2.743	7.943	6.771
$-{\mathrm{SO}}_{3}H$	−18.32	2.80	0.70	−14.82	−8.648	−2.939	7.799	6.575
−CN	−14.47	2.17	0.96	−11.34	−8.628	−2.948	7.779	6.566
$-{\mathrm{CF}}_{3}$	−9.88	2.50	0.79	−6.59	−8.626	−2.746	7.777	6.768
−CHO	−11.63	2.42	0.81	−8.40	−8.639	−3.028	7.790	6.486
−COOH	−11.64	2.45	0.94	−8.25	−8.520	−2.417	7.671	7.097
−NO	−14.42	2.35	4.59	−7.48	−8.267	−3.205	7.418	6.309
−Br	−10.05	2.27	0.81	−6.97	−8.156	−2.352	7.307	7.162
−Cl	−10.23	2.45	0.81	−6.97	−8.222	−2.322	7.373	7.192
−H	−14.87	1.87	1.04	−11.96	−8.339	−2.175	7.490	7.339
−Ph	−11.19	2.33	0.93	−7.93	−7.696	−2.243	6.847	7.271
$-{\mathrm{NHCOCH}}_{3}$	−10.34	2.57	0.80	−6.97	−7.794	−2.141	6.945	7.373
$-{\mathrm{OCH}}_{3}$	−9.58	2.34	−5.72	−12.96	−7.615	−1.686	6.766	7.828
−OH	−9.53	2.30	0.82	−6.41	−7.765	−1.724	6.916	7.790
$-N{\left({\mathrm{CH}}_{3}\right)}_{2}$	−9.32	2.16	0.53	−6.63	−6.943	−1.429	6.094	8.085
$-N{\left({\mathrm{CH}}_{2}{\mathrm{CH}}_{3}\right)}_{2}$	−9.62	2.17	0.62	−6.83	−6.865	−1.410	6.016	8.104
$-N{\left(\mathrm{Pr}\right)}_{2}$	−9.69	2.19	0.63	−6.87	−6.828	−1.373	5.979	8.141
$-{\mathrm{NHCH}}_{3}$	−9.72	2.39	0.72	−6.61	−7.112	−1.379	6.263	8.135
$-{\mathrm{NH}}_{2}$	−9.64	2.33	0.75	−6.56	−7.339	−1.501	6.490	8.013
$-S^{-}$	−15.74	2.24	0.74	−12.76	−2.833	2.359	1.984	11.873
$-O^{-}$	−16.23	2.24	0.73	−13.26	−2.698	3.168	1.849	12.682

**Table 3:** The values of electron density, $\rho_{\left(3,-1\right)}\left(r\right)$ ($e/{\mathrm{au}}^{3}$), Laplacian, $\nabla^{2}\rho_{\left(3,-1\right)}\left(r\right)$ ($e/{\mathrm{au}}^{5}$), delocalization index, DI, and basin populations, $\bar{N}$ (e), for the studied bonds in IsRd derivatives.

Bond	Electron Density						Electron Localization Function		
	$\rho_{\left(3,-1\right)}\left(r\right)$ ($e/{\mathrm{au}}^{3}$)		$\nabla^{2}\rho_{\left(3,-1\right)}\left(r\right)$ ($e/{\mathrm{au}}^{5}$)		DI		Basin	$\bar{N}$ (e)	
	Range	Median	Range	Median	Range	Median		Range	Median
N–H1	0.332–0.336	0.333	−1.735–−1.695	−1.729	0.716–0.750	0.728	V(N,H1)	2.04–2.06	2.06
C4–H2	0.268–0.286	0.282	−1.007–−0.888	−0.974	0.880–0.953	0.911	V(C4,H2)	2.12–2.22	2.17
N–C2	0.298–0.315	0.302	−0.881–−0.839	−0.851	0.937–1.012	0.951	V(N,C2)	1.97–2.10	2.08
N–C1	0.282–0.308	0.304	−0.776–−0.672	−0.765	0.987–1.076	1.060	V(N,C1)	2.08–2.22	2.11
C1–C3	0.258–0.304	0.264	−0.803–−0.615	−0.640	1.005–1.336	1.040	V(C1,C3)	2.23–3.37	2.28
C1–S1	0.206–0.228	0.223	−0.151–0.156	0.086	1.491–1.784	1.723	V(C1,S1)	2.26–2.68	2.55
C2–O	0.407–0.428	0.425	−0.117–0.092	0.054	1.273–1.362	1.340	V(C2,O)	2.25–2.49	2.46
C2–S2	0.189–0.197	0.193	−0.341–−0.308	−0.323	1.014–1.083	1.045	V(C2,S2)	1.90–1.98	1.93
C3–S2	0.184–0.198	0.190	−0.351–−0.281	−0.317	1.086–1.164	1.116	V(C3,S2)	1.66–1.85	1.77
C3–C4	0.286–0.339	0.331	−0.998–−0.747	−0.947	1.157–1.736	1.615	V(C3,C4)	2.59–3.63	3.48

**Table 4:** HOMA values for IsRd derivatives calculated at the DFT(M06-2X)/6-311+G(d,p) computational level.

R	HOMA
$-{\mathrm{SO}}_{2}{\mathrm{CF}}_{3}$	−0.079
$-{\mathrm{NO}}_{2}$	−0.047
$-{\mathrm{SO}}_{3}H$	−0.092
−CN	−0.003
$-{\mathrm{CF}}_{3}$	−0.086
−CHO	−0.051
−COOH	−0.008
−NO	−0.058
−Br	0.049
−Cl	0.066
−H	−0.009
−Ph	0.026
$-{\mathrm{NHCOCH}}_{3}$	0.306
$-{\mathrm{OCH}}_{3}$	0.220
−OH	0.197
$-N{\left({\mathrm{CH}}_{3}\right)}_{2}$	0.368
$-N{\left({\mathrm{CH}}_{2}{\mathrm{CH}}_{3}\right)}_{2}$	0.371
$-N{\left(\mathrm{Pr}\right)}_{2}$	0.373
$-{\mathrm{NHCH}}_{3}$	0.434
$-{\mathrm{NH}}_{2}$	0.403
$-S^{-}$	0.421
$-O^{-}$	0.467

## Data Availability

Data available on request.

## Supplementary Materials

The following supporting information can be downloaded at: https://www.mdpi.com/article/10.3390/molecules28052360/s1, Table S1: The values of ELF basin population, N(e), for all studied IsRd derivatives. Table S2: The values of electron density on bond critical point, ρ_(3,−1)_(r) (e/au^3^), for all studied IsRd derivatives. Table S3: The values of Laplacian on bond critical point, ∇^2^ρ_(3,−1)_(r) (e/au^5^). Table S4: The values of delocalisation index, DI, for all studied IsRd derivatives.
