# Supplementary material for: Exploring the Relationship between Reactivity and Electronic Structure in Isorhodanine Derivatives Using Computer Simulations
Source: Molecules. 2023 Mar 3;28(5):2360. doi: 10.3390/molecules28052360 (PMC10004983; doi:10.3390/molecules28052360)
Supplement: Supplementary file 1 [file molecules-28-02360-s001.zip › molecules-2215273-supplementary.pdf]

Electronic Supporting Information for:

Exploring the relationship between reactivity and electronic structure in isorhodanine derivatives using computer simulations

Michal Michalski [1)], Slawomir Berski [2)]

1. Centre of New Technologies, University of Warsaw, 02-097, Warsaw, Poland
2. Faculty of Chemistry, University of Wroclaw, 50-383, Wroclaw, Poland

Table S1. The values of ELF basin population, $\overline{\text{N}}(\text{e})$, for all studied IsRd derivatives.

R	V(N,H1)	V(C4,H2)	V(N,C2)	V(N,C1)	V(C1,C3)	V(C1,S1)	V(C2,O)	V(C2,S2)	V(C3,S2)	V(C3,C4)	V(C3,C4)
**-SO_2CF_3**	2.05	2.16	2.09	2.09	2.23	2.66	2.49	1.9	1.83	1.77	1.77
**-NO_2**	2.06	2.20	2.09	2.11	2.23	2.63	2.49	1.91	1.82	1.81	1.82
**-SO_3H**	2.05	2.16	2.10	2.08	2.23	2.68	2.48	1.91	1.82	1.76	1.78
**-CN**	2.06	2.12	2.09	2.10	2.25	2.62	2.49	1.91	1.85	1.70	1.70
**-CF_3**	2.06	2.14	2.09	2.10	2.23	2.63	2.48	1.92	1.81	1.76	1.76
**-CHO**	2.06	2.12	2.08	2.14	2.24	2.56	2.48	1.94	1.82	1.71	1.71
**-COOH**	2.06	2.13	2.08	2.13	2.24	2.58	2.47	1.93	1.81	1.77	1.74
**-NO**	2.06	2.18	2.08	2.10	2.23	2.66	2.48	1.91	1.85	1.76	1.72
**-Br**	2.06	2.18	2.10	2.10	2.28	2.58	2.46	1.92	1.77	1.79	1.79
**-Cl**	2.06	2.19	2.10	2.10	2.28	2.58	2.46	1.92	1.78	1.80	1.80
**-H**	2.06	2.12	2.08	2.15	2.23	2.55	2.47	1.93	1.80	1.72	1.72
**-Ph**	2.06	2.12	2.09	2.12	2.27	2.55	2.45	1.94	1.77	1.69	1.79
**-$NHCOCH_3$**	2.06	2.17	2.09	2.11	2.38	2.43	2.45	1.93	1.76	1.75	1.75
**-OCH_3**	2.05	2.21	2.08	2.11	2.32	2.53	2.44	1.92	1.76	1.80	1.80
**-OH**	2.05	2.22	2.08	2.10	2.31	2.54	2.44	1.92	1.76	1.80	1.81
**-$N(CH_3)_2$**	2.05	2.18	2.06	2.12	2.54	2.40	2.41	1.93	1.71	3.39	
**-$N(CH_2CH_3)_2$**	2.05	2.17	2.06	2.12	2.55	2.40	2.41	1.94	1.72	3.38	
**-$N(Pr)_2$**	2.05	2.17	2.05	2.13	2.55	2.40	2.41	1.94	1.72	3.37	
**-$NHCH_3$**	2.05	2.16	2.08	2.11	2.53	2.34	2.42	1.94	1.73	3.40	
**-NH_2**	2.05	2.16	2.09	2.10	2.47	2.37	2.43	1.94	1.74	3.43	
**-S^-**	2.04	2.13	1.98	2.20	2.72	2.35	2.27	1.98	1.68	3.16	
**-O^-**	2.04	2.17	1.97	2.22	3.37	2.26	2.25	1.98	1.66	2.59	

Table S2. The values of electron density on bond critical point, $\rho_{(3,-1)}(r)$ (e/au^3), for all studied IsRd derivatives.

R	N-H1	C4-H2	N-C2	N-C1	C1-C3	C1-S1	C2-O	C2-S2	C3-S2	C3-C4
**-SO_2CF_3**	0.332	0.279	0.302	0.305	0.258	0.228	0.428	0.190	0.195	0.332
**-NO_2**	0.332	0.284	0.301	0.306	0.259	0.226	0.427	0.190	0.194	0.338
**-SO_3H**	0.333	0.281	0.304	0.304	0.258	0.228	0.427	0.190	0.194	0.332
**-CN**	0.333	0.281	0.302	0.305	0.261	0.226	0.427	0.190	0.197	0.331
**-CF_3**	0.333	0.282	0.302	0.305	0.258	0.226	0.426	0.192	0.194	0.334
**-CHO**	0.333	0.281	0.299	0.308	0.259	0.224	0.426	0.194	0.194	0.330
**-COOH**	0.333	0.279	0.299	0.308	0.261	0.225	0.426	0.193	0.193	0.336
**-NO**	0.333	0.281	0.303	0.303	0.259	0.227	0.427	0.189	0.198	0.333
**-Br**	0.333	0.286	0.302	0.305	0.263	0.224	0.425	0.193	0.190	0.333
**-Cl**	0.333	0.286	0.302	0.304	0.264	0.223	0.425	0.193	0.190	0.334
**-H**	0.333	0.281	0.298	0.308	0.261	0.223	0.425	0.193	0.194	0.339
**-Ph**	0.333	0.279	0.301	0.305	0.262	0.222	0.424	0.195	0.190	0.330
**-$NHCOCH_3$**	0.333	0.286	0.300	0.306	0.278	0.217	0.424	0.193	0.190	0.323
**-OCH_3**	0.333	0.284	0.303	0.301	0.272	0.221	0.423	0.192	0.190	0.331
**-OH**	0.333	0.286	0.303	0.302	0.271	0.222	0.424	0.192	0.190	0.333
**-$N(CH_3)_2$**	0.334	0.283	0.305	0.299	0.282	0.214	0.421	0.193	0.188	0.312
**-$N(CH_2CH_3)_2$**	0.334	0.282	0.305	0.299	0.282	0.214	0.421	0.193	0.188	0.310
**-$N(Pr)_2$**	0.334	0.282	0.305	0.298	0.282	0.214	0.420	0.193	0.188	0.310
**-$NHCH_3$**	0.334	0.284	0.302	0.302	0.289	0.213	0.422	0.193	0.190	0.313
**-NH_2**	0.333	0.286	0.301	0.304	0.286	0.214	0.422	0.193	0.190	0.317
**-S^-**	0.336	0.277	0.313	0.285	0.295	0.212	0.408	0.197	0.184	0.301
**-O^-**	0.336	0.268	0.315	0.282	0.304	0.206	0.407	0.195	0.186	0.286

Table S3. The values of Laplacian on bond critical point, $\nabla^2\rho_{(3,-1)}(r)$ (e/au^5), for all studied IsRd derivatives.

R	N-H1	C4-H2	N-C2	N-C1	C1-C3	C1-S1	C2-O	C2-S2	C3-S2	C3-C4
**-SO_2CF_3**	-1.735	-0.948	-0.853	-0.775	-0.619	0.156	0.092	-0.309	-0.342	-0.938
**-NO_2**	-1.734	-0.994	-0.849	-0.774	-0.620	0.141	0.084	-0.312	-0.336	-0.971
**-SO_3H**	-1.732	-0.963	-0.856	-0.774	-0.617	0.155	0.081	-0.313	-0.336	-0.945
**-CN**	-1.733	-0.970	-0.852	-0.769	-0.630	0.131	0.082	-0.313	-0.350	-0.949
**-CF_3**	-1.732	-0.971	-0.850	-0.772	-0.615	0.136	0.076	-0.322	-0.333	-0.956
**-CHO**	-1.733	-0.967	-0.840	-0.775	-0.620	0.091	0.079	-0.329	-0.332	-0.951
**-COOH**	-1.731	-0.957	-0.841	-0.768	-0.631	0.110	0.072	-0.324	-0.331	-0.963
**-NO**	-1.731	-0.969	-0.858	-0.776	-0.620	0.132	0.085	-0.308	-0.351	-0.971
**-Br**	-1.731	-0.998	-0.849	-0.770	-0.638	0.097	0.058	-0.324	-0.315	-0.949
**-Cl**	-1.731	-1.002	-0.850	-0.769	-0.641	0.098	0.059	-0.323	-0.319	-0.955
**-H**	-1.730	-0.973	-0.839	-0.758	-0.629	0.097	0.068	-0.325	-0.332	-0.998
**-Ph**	-1.727	-0.954	-0.846	-0.766	-0.631	0.080	0.049	-0.334	-0.315	-0.943
**-$NHCOCH_3$**	-1.728	-1.007	-0.840	-0.764	-0.697	-0.003	0.044	-0.323	-0.318	-0.906
**-OCH_3**	-1.725	-0.988	-0.855	-0.755	-0.675	0.072	0.035	-0.319	-0.316	-0.954
**-OH**	-1.726	-1.004	-0.855	-0.757	-0.670	0.080	0.040	-0.319	-0.317	-0.965
**-$N(CH_3)_2$**	-1.723	-0.981	-0.857	-0.758	-0.707	-0.037	0.011	-0.323	-0.304	-0.855
**-$N(CH_2CH_3)_2$**	-1.723	-0.975	-0.857	-0.756	-0.705	-0.039	0.008	-0.324	-0.304	-0.848
**-$N(Pr)_2$**	-1.723	-0.976	-0.858	-0.755	-0.705	-0.040	0.007	-0.324	-0.304	-0.847
**-$NHCH_3$**	-1.723	-0.996	-0.844	-0.750	-0.744	-0.087	0.015	-0.321	-0.314	-0.862
**-NH_2**	-1.724	-1.007	-0.842	-0.755	-0.731	-0.062	0.023	-0.323	-0.315	-0.882
**-S^-**	-1.697	-0.934	-0.875	-0.687	-0.765	-0.095	-0.101	-0.341	-0.281	-0.803
**-O^-**	-1.695	-0.888	-0.881	-0.672	-0.803	-0.151	-0.117	-0.332	-0.287	-0.747

Table S4. The values of delocalisation index, DI, for all studied IsRd derivatives.

R	N-H1	C4-H2	N-C2	N-C1	C1-C3	C1-S1	C2-O	C2-S2	C3-S2	C3-C4
**$-SO_2CF_3$**	0.716	0.903	0.950	1.064	1.005	1.780	1.354	1.016	1.149	1.695
**$-NO_2$**	0.723	0.907	0.951	1.064	1.009	1.770	1.353	1.025	1.136	1.683
**$-SO_3H$**	0.725	0.911	0.956	1.059	1.007	1.784	1.362	1.018	1.145	1.697
**-CN**	0.716	0.916	0.952	1.058	1.018	1.765	1.353	1.023	1.154	1.634
**$-CF_3$**	0.729	0.920	0.945	1.062	1.010	1.770	1.351	1.032	1.136	1.697
**-CHO**	0.729	0.921	0.937	1.076	1.020	1.741	1.350	1.040	1.138	1.659
**-COOH**	0.724	0.929	0.942	1.065	1.015	1.745	1.357	1.042	1.129	1.701
**-NO**	0.725	0.907	0.948	1.061	1.014	1.780	1.355	1.014	1.164	1.596
**-Br**	0.727	0.923	0.947	1.063	1.039	1.736	1.347	1.041	1.114	1.667
**-Cl**	0.736	0.918	0.946	1.061	1.041	1.737	1.340	1.040	1.116	1.652
**-H**	0.728	0.953	0.937	1.074	1.015	1.740	1.351	1.049	1.130	1.736
**-Ph**	0.729	0.925	0.945	1.063	1.039	1.722	1.340	1.055	1.116	1.648
**$-NHCOCH_3$**	0.723	0.892	0.951	1.064	1.113	1.636	1.337	1.053	1.116	1.501
**$-OCH_3$**	0.730	0.898	0.956	1.050	1.081	1.704	1.330	1.045	1.115	1.544
**-OH**	0.731	0.913	0.963	1.049	1.073	1.714	1.337	1.045	1.114	1.564
**$-N(CH_3)_2$**	0.725	0.896	0.972	1.043	1.172	1.589	1.326	1.054	1.112	1.394
**$-N(CH_2CH_3)_2$**	0.730	0.891	0.965	1.042	1.175	1.587	1.323	1.053	1.112	1.391
**$-N(Pr)_2$**	0.730	0.890	0.966	1.041	1.177	1.585	1.327	1.054	1.112	1.388
**$-NHCH_3$**	0.736	0.901	0.963	1.050	1.191	1.559	1.326	1.059	1.116	1.393
**$-NH_2$**	0.723	0.912	0.958	1.055	1.166	1.583	1.333	1.061	1.116	1.430
**$-S^-$**	0.750	0.938	1.002	0.999	1.253	1.548	1.273	1.083	1.086	1.356
**$-O^-$**	0.748	0.880	1.012	0.987	1.336	1.491	1.274	1.083	1.102	1.157
